# Supplementary material for: Hot spot prediction in protein-protein interactions by an ensemble system
Source: BMC Syst Biol. 2018 Dec 31;12(Suppl 9):132. doi: 10.1186/s12918-018-0665-8 (PMC6311905; doi:10.1186/s12918-018-0665-8)
Supplement: Supplementary file 2 — All relevant MATLAB code. A simple Matlab implement of our predictor. (ZIP 19,742 kb) [file 12918_2018_665_MOESM2_ESM.zip › Additional file 2/Read ASA/ASA Property/SKEMPI/1HE8A.txt]

 NetSurfP - Protein Surface Accessibility and Secondary Structure Predictions

  

|  |  |  |
| --- | --- | --- |
|  |  | NetSurfP - Protein Surface Accessibility and Secondary Structure Predictions  Technical University of Denmark |

  


---

```
# For publication of results, please cite:
# A generic method for assignment of reliability scores applied to solvent accessibility predictions.
# Bent Petersen, Thomas Nordahl Petersen, Pernille Andersen, Morten Nielsen and Claus Lundegaard
# BMC Structural Biology 2009, 9:51 doi:10.1186/1472-6807-9-51
#
# Column 1: Class assignment - B for buried or E for Exposed - Threshold: 25% exposure, but not based on RSA
# Column 2: Amino acid
# Column 3: Sequence name
# Column 4: Amino acid number
# Column 5: Relative Surface Accessibility - RSA
# Column 6: Absolute Surface Accessibility
# Column 7: Z-fit score for RSA prediction
# Column 8: Probability for Alpha-Helix
# Column 9: Probability for Beta-strand
# Column 10: Probability for Coil
E S  1HE8A                  1    0.897 105.175   0.531   0.016   0.005   0.979
E E  1HE8A                  2    0.580 101.361   0.688   0.717   0.014   0.269
E E  1HE8A                  3    0.442  77.287  -0.127   0.802   0.014   0.185
E S  1HE8A                  4    0.494  57.909   0.380   0.802   0.014   0.185
E Q  1HE8A                  5    0.437  78.030   0.859   0.831   0.044   0.125
E A  1HE8A                  6    0.387  42.647   1.227   0.831   0.044   0.125
B F  1HE8A                  7    0.114  22.920   0.549   0.831   0.044   0.125
E Q  1HE8A                  8    0.297  53.098   0.096   0.831   0.044   0.125
E R  1HE8A                  9    0.439 100.554   0.325   0.831   0.044   0.125
B Q  1HE8A                 10    0.285  50.883   0.499   0.831   0.044   0.125
B L  1HE8A                 11    0.040   7.269   0.591   0.751   0.050   0.199
B T  1HE8A                 12    0.212  29.404  -0.544   0.751   0.050   0.199
E A  1HE8A                 13    0.329  36.212  -0.058   0.701   0.107   0.192
B L  1HE8A                 14    0.058  10.583  -0.449   0.701   0.107   0.192
B I  1HE8A                 15    0.099  18.241   0.640   0.502   0.102   0.396
E G  1HE8A                 16    0.444  34.943  -1.072   0.113   0.087   0.800
E Y  1HE8A                 17    0.339  72.551   0.894   0.018   0.047   0.935
E D  1HE8A                 18    0.516  74.384  -0.562   0.018   0.047   0.935
B V  1HE8A                 19    0.108  16.569   0.645   0.191   0.086   0.723
B T  1HE8A                 20    0.274  37.990   0.405   0.278   0.093   0.628
E D  1HE8A                 21    0.426  61.430   0.682   0.386   0.097   0.517
B V  1HE8A                 22    0.145  22.302   0.122   0.386   0.097   0.517
E S  1HE8A                 23    0.312  36.578  -0.267   0.386   0.097   0.517
E N  1HE8A                 24    0.555  81.193  -0.508   0.268   0.043   0.689
E V  1HE8A                 25    0.416  63.924  -0.762   0.113   0.043   0.844
E H  1HE8A                 26    0.476  86.584   0.466   0.018   0.019   0.964
E D  1HE8A                 27    0.381  54.844  -0.053   0.018   0.019   0.964
E D  1HE8A                 28    0.697 100.495   0.316   0.694   0.003   0.303
E E  1HE8A                 29    0.373  65.181   1.188   0.938   0.007   0.055
B L  1HE8A                 30    0.051   9.393   1.050   0.975   0.003   0.022
E E  1HE8A                 31    0.351  61.285   1.533   0.970   0.001   0.030
E F  1HE8A                 32    0.525 105.367   1.074   0.970   0.001   0.030
B T  1HE8A                 33    0.098  13.620   0.775   0.970   0.001   0.030
B R  1HE8A                 34    0.176  40.350   0.513   0.970   0.001   0.030
E R  1HE8A                 35    0.331  75.845   0.652   0.970   0.001   0.030
E G  1HE8A                 36    0.424  33.385   1.517   0.970   0.001   0.030
B L  1HE8A                 37    0.073  13.366   0.630   0.970   0.001   0.030
B V  1HE8A                 38    0.144  22.117   0.739   0.988   0.000   0.012
E T  1HE8A                 39    0.498  69.128   1.191   0.988   0.000   0.012
E P  1HE8A                 40    0.286  40.583   1.203   0.988   0.000   0.012
B R  1HE8A                 41    0.112  25.740   0.357   0.970   0.001   0.030
E M  1HE8A                 42    0.373  74.637   0.871   0.970   0.001   0.030
E A  1HE8A                 43    0.633  69.746   0.652   0.923   0.002   0.076
E E  1HE8A                 44    0.478  83.489   0.618   0.782   0.003   0.216
B V  1HE8A                 45    0.186  28.634   0.920   0.502   0.002   0.495
E A  1HE8A                 46    0.607  66.891   0.639   0.522   0.016   0.462
E S  1HE8A                 47    0.403  47.208  -0.904   0.561   0.047   0.393
E R  1HE8A                 48    0.397  90.982  -0.340   0.354   0.048   0.598
E D  1HE8A                 49    0.636  91.590  -0.611   0.268   0.043   0.689
B P  1HE8A                 50    0.300  42.527   0.598   0.561   0.047   0.393
E K  1HE8A                 51    0.315  64.734  -0.300   0.561   0.047   0.393
B L  1HE8A                 52    0.249  45.574   0.408   0.660   0.049   0.291
B Y  1HE8A                 53    0.302  64.559  -1.008   0.522   0.016   0.462
E A  1HE8A                 54    0.453  49.899  -0.431   0.339   0.016   0.645
B M  1HE8A                 55    0.144  28.714   0.624   0.113   0.043   0.844
E H  1HE8A                 56    0.366  66.539   0.020   0.018   0.047   0.935
E P  1HE8A                 57    0.410  58.122  -1.039   0.018   0.047   0.935
E W  1HE8A                 58    0.358  86.171  -0.507   0.018   0.088   0.893
B V  1HE8A                 59    0.084  12.957  -0.237   0.019   0.141   0.840
E T  1HE8A                 60    0.481  66.715  -0.848   0.020   0.205   0.775
B S  1HE8A                 61    0.239  27.964  -0.868   0.020   0.205   0.775
E K  1HE8A                 62    0.525 108.054  -0.046   0.019   0.141   0.840
E P  1HE8A                 63    0.624  88.617  -0.941   0.019   0.141   0.840
E L  1HE8A                 64    0.319  58.482   0.321   0.053   0.043   0.903
B P  1HE8A                 65    0.229  32.523  -0.877   0.181   0.016   0.803
E E  1HE8A                 66    0.678 118.394   0.505   0.430   0.016   0.555
E Y  1HE8A                 67    0.383  81.890   0.192   0.522   0.016   0.462
B L  1HE8A                 68    0.067  12.249   0.394   0.561   0.047   0.393
E W  1HE8A                 69    0.462 111.063   0.641   0.502   0.102   0.396
E K  1HE8A                 70    0.566 116.406   0.651   0.502   0.102   0.396
E K  1HE8A                 71    0.433  89.027   0.876   0.428   0.171   0.402
B I  1HE8A                 72    0.072  13.376  -0.656   0.307   0.165   0.527
E A  1HE8A                 73    0.482  53.094   0.061   0.113   0.087   0.800
E N  1HE8A                 74    0.540  79.085  -0.549   0.018   0.088   0.893
E N  1HE8A                 75    0.271  39.616  -0.633   0.004   0.138   0.858
B C  1HE8A                 76    0.241  33.879   1.084   0.004   0.616   0.381
B I  1HE8A                 77    0.052   9.565   0.196   0.001   0.900   0.099
B F  1HE8A                 78    0.067  13.527  -0.437   0.001   0.959   0.040
B I  1HE8A                 79    0.028   5.180   0.241   0.001   0.959   0.040
B K  1HE8A                 80    0.131  27.029   0.916   0.001   0.959   0.040
B I  1HE8A                 81    0.059  11.007  -0.026   0.001   0.959   0.040
E H  1HE8A                 82    0.335  60.955   0.203   0.003   0.718   0.279
E R  1HE8A                 83    0.429  98.218  -1.036   0.004   0.197   0.799
E S  1HE8A                 84    0.559  65.573  -1.649   0.018   0.088   0.893
E T  1HE8A                 85    0.642  89.087   0.643   0.021   0.279   0.699
E T  1HE8A                 86    0.311  43.122   0.157   0.003   0.718   0.279
E S  1HE8A                 87    0.431  50.537   1.063   0.011   0.918   0.071
B Q  1HE8A                 88    0.216  38.542   1.130   0.001   0.959   0.040
B T  1HE8A                 89    0.265  36.714  -0.001   0.001   0.959   0.040
B I  1HE8A                 90    0.110  20.294   0.585   0.001   0.900   0.099
E K  1HE8A                 91    0.528 108.692   0.990   0.003   0.718   0.279
B V  1HE8A                 92    0.062   9.499   0.925   0.004   0.420   0.576
E S  1HE8A                 93    0.284  33.250   0.579   0.004   0.085   0.910
B P  1HE8A                 94    0.159  22.505  -0.291   0.058   0.017   0.925
E D  1HE8A                 95    0.519  74.773   0.332   0.406   0.004   0.590
E D  1HE8A                 96    0.264  37.999   0.286   0.406   0.004   0.590
B T  1HE8A                 97    0.227  31.471   0.951   0.321   0.003   0.675
B P  1HE8A                 98    0.032   4.527  -0.092   0.923   0.002   0.076
E G  1HE8A                 99    0.371  29.213   1.008   0.970   0.001   0.030
E A  1HE8A                100    0.430  47.375   1.845   0.970   0.001   0.030
B I  1HE8A                101    0.043   8.029   0.560   0.970   0.001   0.030
B L  1HE8A                102    0.045   8.258   0.774   0.970   0.001   0.030
E Q  1HE8A                103    0.448  80.084   1.248   0.970   0.001   0.030
E S  1HE8A                104    0.325  38.090   1.066   0.970   0.001   0.030
B F  1HE8A                105    0.056  11.179   0.507   0.970   0.001   0.030
B F  1HE8A                106    0.085  17.039  -0.130   0.970   0.001   0.030
E T  1HE8A                107    0.417  57.810   1.195   0.988   0.000   0.012
B K  1HE8A                108    0.237  48.710   0.377   0.970   0.001   0.030
B M  1HE8A                109    0.136  27.274   0.265   0.970   0.001   0.030
E A  1HE8A                110    0.425  46.835   0.165   0.923   0.002   0.076
E K  1HE8A                111    0.620 127.493   0.299   0.782   0.003   0.216
E K  1HE8A                112    0.374  77.035  -0.120   0.502   0.002   0.495
E K  1HE8A                113    0.487 100.258  -1.015   0.176   0.004   0.820
E S  1HE8A                114    0.427  49.998   0.034   0.268   0.043   0.689
E L  1HE8A                115    0.291  53.245  -0.564   0.386   0.097   0.517
B M  1HE8A                116    0.170  34.017  -0.977   0.278   0.093   0.628
E D  1HE8A                117    0.546  78.736  -0.887   0.191   0.086   0.723
E I  1HE8A                118    0.353  65.231  -0.318   0.113   0.087   0.800
E P  1HE8A                119    0.459  65.175  -0.777   0.052   0.084   0.864
E E  1HE8A                120    0.559  97.710  -0.529   0.056   0.142   0.802
E S  1HE8A                121    0.644  75.430  -1.363   0.052   0.084   0.864
E Q  1HE8A                122    0.532  95.087   0.031   0.052   0.084   0.864
E S  1HE8A                123    0.527  61.753   0.654   0.018   0.019   0.964
E E  1HE8A                124    0.375  65.530  -0.260   0.018   0.047   0.935
E Q  1HE8A                125    0.487  86.960  -0.142   0.020   0.205   0.775
E D  1HE8A                126    0.335  48.245   0.097   0.022   0.552   0.426
B F  1HE8A                127    0.116  23.321   1.292   0.018   0.846   0.136
B V  1HE8A                128    0.147  22.532   0.944   0.011   0.918   0.071
B L  1HE8A                129    0.047   8.551   1.115   0.011   0.918   0.071
B R  1HE8A                130    0.239  54.662   1.118   0.001   0.900   0.099
B V  1HE8A                131    0.066  10.175  -0.142   0.002   0.816   0.182
B C  1HE8A                132    0.249  34.974   0.324   0.004   0.514   0.481
E G  1HE8A                133    0.316  24.830  -0.978   0.005   0.262   0.733
E R  1HE8A                134    0.312  71.471  -0.009   0.022   0.359   0.619
E D  1HE8A                135    0.419  60.378  -0.497   0.022   0.552   0.426
B E  1HE8A                136    0.187  32.721  -0.184   0.023   0.655   0.322
B Y  1HE8A                137    0.186  39.770   0.035   0.023   0.655   0.322
B L  1HE8A                138    0.115  21.020  -0.314   0.023   0.655   0.322
B V  1HE8A                139    0.200  30.679   0.340   0.022   0.552   0.426
E G  1HE8A                140    0.481  37.855  -0.697   0.019   0.141   0.840
E E  1HE8A                141    0.742 129.575  -0.790   0.005   0.015   0.979
B T  1HE8A                142    0.220  30.569   1.317   0.018   0.088   0.893
E P  1HE8A                143    0.305  43.350   0.314   0.019   0.141   0.840
B I  1HE8A                144    0.093  17.113  -0.689   0.021   0.279   0.699
B K  1HE8A                145    0.188  38.672  -0.174   0.125   0.227   0.648
E N  1HE8A                146    0.360  52.689   0.386   0.199   0.152   0.649
B F  1HE8A                147    0.079  15.795   0.414   0.649   0.163   0.188
E Q  1HE8A                148    0.518  92.586   1.192   0.779   0.100   0.120
B W  1HE8A                149    0.201  48.341   0.709   0.870   0.077   0.053
B V  1HE8A                150    0.035   5.349  -0.192   0.950   0.028   0.022
B R  1HE8A                151    0.216  49.487   0.925   0.938   0.007   0.055
E H  1HE8A                152    0.507  92.223   1.673   0.938   0.007   0.055
B C  1HE8A                153    0.053   7.399   0.330   0.938   0.007   0.055
B L  1HE8A                154    0.161  29.497   0.181   0.879   0.010   0.111
E K  1HE8A                155    0.647 133.006   0.697   0.802   0.014   0.185
E N  1HE8A                156    0.543  79.495   0.227   0.406   0.004   0.590
E G  1HE8A                157    0.665  52.328  -1.642   0.016   0.005   0.979
E E  1HE8A                158    0.497  86.896   0.250   0.018   0.019   0.964
E E  1HE8A                159    0.541  94.565   0.451   0.018   0.047   0.935
B I  1HE8A                160    0.079  14.689  -0.160   0.005   0.045   0.951
E H  1HE8A                161    0.518  94.188   0.633   0.004   0.197   0.799
B V  1HE8A                162    0.100  15.385   0.579   0.004   0.514   0.481
B V  1HE8A                163    0.218  33.430   0.553   0.021   0.756   0.223
B L  1HE8A                164    0.155  28.454  -0.131   0.021   0.756   0.223
E D  1HE8A                165    0.351  50.565  -1.383   0.023   0.655   0.322
E T  1HE8A                166    0.343  47.505  -0.407   0.005   0.336   0.660
E P  1HE8A                167    0.435  61.670  -0.551   0.004   0.085   0.910
E P  1HE8A                168    0.404  57.356  -0.896   0.005   0.015   0.979
E D  1HE8A                169    0.689  99.328  -0.650   0.005   0.015   0.979
B P  1HE8A                170    0.260  36.837  -1.723   0.018   0.019   0.964
E A  1HE8A                171    0.506  55.761  -0.527   0.016   0.005   0.979
E L  1HE8A                172    0.439  80.436  -1.073   0.018   0.019   0.964
E D  1HE8A                173    0.703 101.273  -0.060   0.058   0.017   0.925
E E  1HE8A                174    0.638 111.511   0.373   0.053   0.043   0.903
E V  1HE8A                175    0.513  78.894  -0.665   0.052   0.084   0.864
E R  1HE8A                176    0.378  86.631  -0.299   0.021   0.279   0.699
E K  1HE8A                177    0.356  73.250   0.355   0.021   0.451   0.528
B E  1HE8A                178    0.264  46.191   0.136   0.022   0.552   0.426
B S  1HE8A                179    0.267  31.246  -0.931   0.074   0.484   0.442
B L  1HE8A                180    0.237  43.340  -0.940   0.064   0.216   0.721
B W  1HE8A                181    0.271  65.200  -0.327   0.064   0.216   0.721
E D  1HE8A                182    0.543  78.203   0.681   0.064   0.216   0.721
B C  1HE8A                183    0.128  17.915  -0.349   0.019   0.141   0.840
E D  1HE8A                184    0.720 103.824  -0.154   0.018   0.047   0.935
E R  1HE8A                185    0.522 119.515   0.161   0.004   0.085   0.910
E K  1HE8A                186    0.451  92.750   1.036   0.004   0.420   0.576
B F  1HE8A                187    0.050  10.015   0.390   0.003   0.718   0.279
E R  1HE8A                188    0.250  57.319   1.068   0.001   0.900   0.099
B V  1HE8A                189    0.022   3.381   0.647   0.001   0.959   0.040
E K  1HE8A                190    0.232  47.743   1.676   0.001   0.959   0.040
B I  1HE8A                191    0.027   4.976   0.433   0.001   0.959   0.040
E R  1HE8A                192    0.275  62.929   0.251   0.001   0.900   0.099
B G  1HE8A                193    0.158  12.458  -1.567   0.004   0.616   0.381
B I  1HE8A                194    0.114  21.053  -0.516   0.004   0.420   0.576
E D  1HE8A                195    0.496  71.488  -0.433   0.005   0.336   0.660
B I  1HE8A                196    0.099  18.389   0.138   0.004   0.138   0.858
E P  1HE8A                197    0.322  45.749  -0.665   0.004   0.085   0.910
B V  1HE8A                198    0.291  44.711  -1.303   0.019   0.141   0.840
E L  1HE8A                199    0.361  66.154  -0.487   0.004   0.197   0.799
B P  1HE8A                200    0.234  33.247  -0.375   0.005   0.336   0.660
B L  1HE8A                201    0.066  12.140  -0.459   0.003   0.718   0.279
E T  1HE8A                202    0.263  36.423   0.710   0.001   0.900   0.099
B V  1HE8A                203    0.037   5.687  -0.046   0.001   0.959   0.040
B F  1HE8A                204    0.077  15.494  -0.266   0.001   0.959   0.040
B V  1HE8A                205    0.041   6.240  -0.300   0.001   0.959   0.040
B E  1HE8A                206    0.167  29.157   0.385   0.001   0.959   0.040
B A  1HE8A                207    0.032   3.548  -0.492   0.001   0.959   0.040
B N  1HE8A                208    0.050   7.261  -0.236   0.001   0.959   0.040
B I  1HE8A                209    0.047   8.639   0.341   0.001   0.900   0.099
B Q  1HE8A                210    0.079  14.163  -0.235   0.002   0.816   0.182
B H  1HE8A                211    0.137  24.993   0.585   0.004   0.616   0.381
B G  1HE8A                212    0.173  13.647  -1.700   0.020   0.205   0.775
E Q  1HE8A                213    0.418  74.583  -1.301   0.019   0.141   0.840
E Q  1HE8A                214    0.422  75.441   0.165   0.021   0.451   0.528
E V  1HE8A                215    0.402  61.803  -0.010   0.021   0.451   0.528
B L  1HE8A                216    0.131  23.986  -0.347   0.066   0.296   0.638
B C  1HE8A                217    0.166  23.363  -0.420   0.056   0.142   0.802
E Q  1HE8A                218    0.595 106.356   1.047   0.056   0.142   0.802
E R  1HE8A                219    0.491 112.370  -0.265   0.064   0.216   0.721
E R  1HE8A                220    0.413  94.531  -0.502   0.066   0.296   0.638
E T  1HE8A                221    0.490  67.963  -0.774   0.066   0.296   0.638
E S  1HE8A                222    0.404  47.372   0.189   0.020   0.205   0.775
E P  1HE8A                223    0.537  76.144  -0.284   0.056   0.142   0.802
E K  1HE8A                224    0.554 113.876  -0.034   0.052   0.084   0.864
E P  1HE8A                225    0.466  66.168  -1.159   0.053   0.043   0.903
E F  1HE8A                226    0.492  98.724  -1.622   0.053   0.043   0.903
E T  1HE8A                227    0.327  45.397  -1.946   0.052   0.084   0.864
E E  1HE8A                228    0.630 109.991  -1.449   0.052   0.084   0.864
E E  1HE8A                229    0.410  71.644  -0.796   0.018   0.088   0.893
B V  1HE8A                230    0.234  35.935  -1.583   0.019   0.141   0.840
E L  1HE8A                231    0.389  71.263   0.435   0.021   0.279   0.699
B W  1HE8A                232    0.172  41.438  -0.882   0.022   0.359   0.619
E N  1HE8A                233    0.462  67.637  -0.374   0.022   0.359   0.619
E V  1HE8A                234    0.280  42.990  -0.165   0.022   0.552   0.426
E W  1HE8A                235    0.282  67.821   0.471   0.021   0.756   0.223
B L  1HE8A                236    0.129  23.657   1.085   0.021   0.756   0.223
E E  1HE8A                237    0.514  89.708  -0.443   0.021   0.756   0.223
B F  1HE8A                238    0.165  33.035   0.987   0.022   0.359   0.619
E S  1HE8A                239    0.664  77.821  -1.268   0.019   0.141   0.840
B I  1HE8A                240    0.138  25.474   0.783   0.005   0.045   0.951
E K  1HE8A                241    0.507 104.393   0.481   0.018   0.047   0.935
B I  1HE8A                242    0.120  22.108   0.067   0.018   0.019   0.964
E K  1HE8A                243    0.694 142.653   0.434   0.115   0.016   0.868
E D  1HE8A                244    0.639  92.094  -0.432   0.246   0.004   0.750
B L  1HE8A                245    0.174  31.896   0.274   0.406   0.004   0.590
E P  1HE8A                246    0.358  50.843   0.659   0.782   0.003   0.216
E K  1HE8A                247    0.512 105.216   0.423   0.923   0.002   0.076
E G  1HE8A                248    0.367  28.851   0.359   0.970   0.001   0.030
B A  1HE8A                249    0.045   4.981  -0.467   0.970   0.001   0.030
B L  1HE8A                250    0.247  45.262   0.663   0.970   0.001   0.030
E L  1HE8A                251    0.327  59.892   0.366   0.923   0.002   0.076
B N  1HE8A                252    0.087  12.722   0.373   0.923   0.002   0.076
B L  1HE8A                253    0.041   7.470  -0.649   0.879   0.010   0.111
E Q  1HE8A                254    0.406  72.565  -0.157   0.717   0.014   0.269
E I  1HE8A                255    0.419  77.478  -0.330   0.522   0.016   0.462
E Y  1HE8A                256    0.299  63.854   0.279   0.257   0.016   0.727
B C  1HE8A                257    0.245  34.440  -1.430   0.115   0.016   0.868
E K  1HE8A                258    0.433  88.965  -0.735   0.058   0.017   0.925
E V  1HE8A                259    0.467  71.701  -0.854   0.113   0.043   0.844
E R  1HE8A                260    0.445 101.859   0.753   0.184   0.043   0.773
B L  1HE8A                261    0.138  25.359  -0.371   0.339   0.016   0.645
B L  1HE8A                262    0.161  29.516   0.218   0.522   0.016   0.462
E Y  1HE8A                263    0.505 107.940  -0.333   0.717   0.014   0.269
E Y  1HE8A                264    0.370  78.984  -0.223   0.660   0.049   0.291
B V  1HE8A                265    0.205  31.493  -0.779   0.660   0.049   0.291
E N  1HE8A                266    0.330  48.327  -0.588   0.522   0.016   0.462
B L  1HE8A                267    0.285  52.092   0.168   0.622   0.015   0.363
B L  1HE8A                268    0.189  34.606   0.420   0.717   0.014   0.269
B L  1HE8A                269    0.127  23.199  -0.065   0.879   0.010   0.111
B I  1HE8A                270    0.096  17.723   0.127   0.938   0.007   0.055
E D  1HE8A                271    0.377  54.268   0.554   0.923   0.002   0.076
B H  1HE8A                272    0.160  29.068   0.499   0.923   0.002   0.076
B R  1HE8A                273    0.195  44.632   0.274   0.970   0.001   0.030
B F  1HE8A                274    0.247  49.653   0.631   0.970   0.001   0.030
B L  1HE8A                275    0.107  19.665   0.465   0.970   0.001   0.030
B L  1HE8A                276    0.056  10.290  -0.083   0.938   0.007   0.055
E R  1HE8A                277    0.385  88.096   0.342   0.879   0.010   0.111
E R  1HE8A                278    0.538 123.179   0.189   0.717   0.014   0.269
B G  1HE8A                279    0.278  21.879  -0.006   0.257   0.016   0.727
E E  1HE8A                280    0.446  77.986  -0.585   0.430   0.016   0.555
E Y  1HE8A                281    0.444  94.797  -0.510   0.622   0.015   0.363
B V  1HE8A                282    0.196  30.202   0.260   0.717   0.014   0.269
B L  1HE8A                283    0.166  30.321  -1.145   0.717   0.014   0.269
E H  1HE8A                284    0.453  82.328  -0.658   0.522   0.016   0.462
E M  1HE8A                285    0.448  89.625  -0.270   0.430   0.016   0.555
B W  1HE8A                286    0.220  53.030   0.578   0.181   0.016   0.803
E Q  1HE8A                287    0.401  71.565  -0.890   0.339   0.016   0.645
E I  1HE8A                288    0.551 101.935  -0.896   0.622   0.015   0.363
E S  1HE8A                289    0.314  36.848   0.263   0.717   0.014   0.269
B N  1HE8A                290    0.096  14.054   0.168   0.802   0.014   0.185
E A  1HE8A                291    0.351  38.647   0.386   0.717   0.014   0.269
E D  1HE8A                292    0.394  56.718   0.889   0.717   0.014   0.269
B K  1HE8A                293    0.067  13.700   0.564   0.751   0.050   0.199
B L  1HE8A                294    0.040   7.379  -0.971   0.649   0.163   0.188
E T  1HE8A                295    0.401  55.577   1.011   0.594   0.285   0.121
B S  1HE8A                296    0.168  19.748  -0.906   0.373   0.401   0.226
B A  1HE8A                297    0.129  14.238   0.958   0.125   0.227   0.648
E T  1HE8A                298    0.574  79.614  -0.215   0.018   0.088   0.893
B N  1HE8A                299    0.274  40.070   0.006   0.018   0.019   0.964
E P  1HE8A                300    0.543  76.995  -1.316   0.016   0.005   0.979
E D  1HE8A                301    0.495  71.286  -0.341   0.016   0.005   0.979
E K  1HE8A                302    0.605 124.510   0.133   0.782   0.003   0.216
E E  1HE8A                303    0.558  97.500   0.502   0.782   0.003   0.216
B N  1HE8A                304    0.198  29.046   0.447   0.782   0.003   0.216
B S  1HE8A                305    0.200  23.475   0.916   0.970   0.001   0.030
E M  1HE8A                306    0.556 111.216   1.255   0.988   0.000   0.012
B S  1HE8A                307    0.235  27.601   1.383   0.988   0.000   0.012
B I  1HE8A                308    0.014   2.608   1.622   0.988   0.000   0.012
B S  1HE8A                309    0.216  25.327   1.230   0.988   0.000   0.012
E I  1HE8A                310    0.377  69.653   1.048   0.970   0.001   0.030
B L  1HE8A                311    0.067  12.304   0.907   0.970   0.001   0.030
B L  1HE8A                312    0.020   3.754   1.097   0.970   0.001   0.030
E D  1HE8A                313    0.398  57.352   1.286   0.970   0.001   0.030
E N  1HE8A                314    0.471  68.954   0.770   0.923   0.002   0.076
B Y  1HE8A                315    0.097  20.814  -0.135   0.858   0.002   0.139
B C  1HE8A                316    0.172  24.079  -1.738   0.600   0.003   0.397
E H  1HE8A                317    0.512  93.115  -0.701   0.321   0.003   0.675
E P  1HE8A                318    0.449  63.642  -1.254   0.246   0.004   0.750
B I  1HE8A                319    0.204  37.721   0.200   0.109   0.005   0.886
E S  1HE8A                320    0.511  59.889  -1.197   0.058   0.017   0.925
E A  1HE8A                321    0.454  50.053  -1.283   0.115   0.016   0.868
B L  1HE8A                322    0.142  25.909  -0.506   0.109   0.005   0.886
E D  1HE8A                323    0.393  56.559   0.136   0.109   0.005   0.886
B V  1HE8A                324    0.113  17.414  -0.033   0.858   0.002   0.139
E G  1HE8A                325    0.333  26.239  -0.709   0.858   0.002   0.139
E L  1HE8A                326    0.288  52.660   0.620   0.879   0.010   0.111
B T  1HE8A                327    0.017   2.386   1.016   0.938   0.007   0.055
B M  1HE8A                328    0.032   6.323   0.424   0.938   0.007   0.055
E Q  1HE8A                329    0.359  64.171   0.603   0.879   0.010   0.111
B L  1HE8A                330    0.074  13.641  -0.274   0.782   0.003   0.216
B L  1HE8A                331    0.043   7.837  -0.076   0.694   0.003   0.303
B D  1HE8A                332    0.183  26.414   0.149   0.246   0.004   0.750
E C  1HE8A                333    0.411  57.718  -0.377   0.109   0.005   0.886
E N  1HE8A                334    0.555  81.296   0.084   0.053   0.005   0.942
B F  1HE8A                335    0.081  16.257   0.291   0.016   0.005   0.979
E S  1HE8A                336    0.613  71.855   0.571   0.016   0.005   0.979
B D  1HE8A                337    0.262  37.769  -0.105   0.694   0.003   0.303
E E  1HE8A                338    0.607 106.095  -0.068   0.970   0.001   0.030
E N  1HE8A                339    0.311  45.574   1.134   0.970   0.001   0.030
B V  1HE8A                340    0.027   4.211   0.408   0.970   0.001   0.030
B R  1HE8A                341    0.135  31.007   1.362   0.988   0.000   0.012
E A  1HE8A                342    0.451  49.700   1.431   0.988   0.000   0.012
B I  1HE8A                343    0.111  20.498   1.076   0.988   0.000   0.012
B A  1HE8A                344    0.019   2.116   0.788   0.988   0.000   0.012
B V  1HE8A                345    0.122  18.736   0.213   0.970   0.001   0.030
E Q  1HE8A                346    0.507  90.497   1.341   0.970   0.001   0.030
B K  1HE8A                347    0.155  31.781   1.032   0.923   0.002   0.076
B L  1HE8A                348    0.036   6.537  -0.496   0.858   0.002   0.139
E E  1HE8A                349    0.525  91.717   0.882   0.694   0.003   0.303
E S  1HE8A                350    0.696  81.571   1.445   0.321   0.003   0.675
B L  1HE8A                351    0.128  23.455   1.344   0.016   0.005   0.979
E E  1HE8A                352    0.504  88.084   0.307   0.003   0.003   0.994
E D  1HE8A                353    0.327  47.077  -0.172   0.782   0.003   0.216
E D  1HE8A                354    0.520  74.975  -0.303   0.923   0.002   0.076
B D  1HE8A                355    0.219  31.601   1.029   0.923   0.002   0.076
B V  1HE8A                356    0.040   6.209   0.058   0.970   0.001   0.030
B L  1HE8A                357    0.180  32.921   0.948   0.970   0.001   0.030
B H  1HE8A                358    0.241  43.892  -0.052   0.970   0.001   0.030
B Y  1HE8A                359    0.055  11.668   0.827   0.970   0.001   0.030
B L  1HE8A                360    0.070  12.872  -0.013   0.970   0.001   0.030
B L  1HE8A                361    0.192  35.210  -0.493   0.970   0.001   0.030
B Q  1HE8A                362    0.088  15.788  -0.105   0.970   0.001   0.030
B L  1HE8A                363    0.030   5.475   0.209   0.988   0.000   0.012
B V  1HE8A                364    0.067  10.344   0.445   0.970   0.001   0.030
E Q  1HE8A                365    0.223  39.756   0.098   0.970   0.001   0.030
B A  1HE8A                366    0.181  20.001   0.401   0.923   0.002   0.076
B V  1HE8A                367    0.050   7.670   0.433   0.782   0.003   0.216
E K  1HE8A                368    0.334  68.601   0.242   0.406   0.004   0.590
B F  1HE8A                369    0.322  64.706  -0.528   0.109   0.005   0.886
E E  1HE8A                370    0.470  82.144  -0.294   0.058   0.017   0.925
E P  1HE8A                371    0.454  64.423  -0.652   0.184   0.043   0.773
E Y  1HE8A                372    0.434  92.810  -0.686   0.257   0.016   0.727
E H  1HE8A                373    0.422  76.816  -1.408   0.430   0.016   0.555
E D  1HE8A                374    0.327  47.178  -0.799   0.622   0.015   0.363
E S  1HE8A                375    0.315  36.895  -0.218   0.802   0.014   0.185
E A  1HE8A                376    0.333  36.686   1.309   0.923   0.002   0.076
B L  1HE8A                377    0.045   8.331   0.089   0.970   0.001   0.030
B A  1HE8A                378    0.052   5.686   0.331   0.970   0.001   0.030
E R  1HE8A                379    0.394  90.249   1.264   0.988   0.000   0.012
B F  1HE8A                380    0.120  23.984   1.361   0.988   0.000   0.012
B L  1HE8A                381    0.027   4.925   0.573   0.988   0.000   0.012
B L  1HE8A                382    0.083  15.197   0.454   0.988   0.000   0.012
E K  1HE8A                383    0.415  85.304   1.614   0.970   0.001   0.030
B R  1HE8A                384    0.132  30.342   0.872   0.970   0.001   0.030
B G  1HE8A                385    0.029   2.267  -1.098   0.858   0.002   0.139
E L  1HE8A                386    0.318  58.244  -0.727   0.600   0.003   0.397
E R  1HE8A                387    0.591 135.362  -0.074   0.321   0.003   0.675
B N  1HE8A                388    0.172  25.239  -0.487   0.053   0.005   0.942
E K  1HE8A                389    0.394  80.943  -0.040   0.246   0.004   0.750
E R  1HE8A                390    0.446 102.065   0.397   0.522   0.016   0.462
B I  1HE8A                391    0.085  15.669   0.543   0.717   0.014   0.269
B G  1HE8A                392    0.040   3.156  -0.982   0.717   0.014   0.269
B H  1HE8A                393    0.149  27.030  -0.512   0.879   0.010   0.111
B F  1HE8A                394    0.190  38.153   0.559   0.858   0.002   0.139
B L  1HE8A                395    0.036   6.647  -0.593   0.923   0.002   0.076
B F  1HE8A                396    0.082  16.377  -0.028   0.923   0.002   0.076
B W  1HE8A                397    0.098  23.689  -0.447   0.923   0.002   0.076
B F  1HE8A                398    0.110  22.037  -0.990   0.858   0.002   0.139
B L  1HE8A                399    0.092  16.809  -0.307   0.782   0.003   0.216
B R  1HE8A                400    0.228  52.281  -0.339   0.782   0.003   0.216
E S  1HE8A                401    0.307  36.004  -0.924   0.622   0.015   0.363
E E  1HE8A                402    0.467  81.532  -1.002   0.430   0.016   0.555
E I  1HE8A                403    0.449  83.139  -0.943   0.268   0.043   0.689
E A  1HE8A                404    0.396  43.672  -0.905   0.113   0.043   0.844
E Q  1HE8A                405    0.468  83.638  -1.135   0.058   0.017   0.925
E S  1HE8A                406    0.512  59.971  -0.411   0.018   0.019   0.964
E R  1HE8A                407    0.630 144.362  -0.325   0.782   0.003   0.216
E H  1HE8A                408    0.601 109.322  -0.250   0.782   0.003   0.216
B Y  1HE8A                409    0.142  30.239  -0.003   0.858   0.002   0.139
B Q  1HE8A                410    0.240  42.918  -0.287   0.923   0.002   0.076
E Q  1HE8A                411    0.539  96.301   0.393   0.923   0.002   0.076
E R  1HE8A                412    0.328  75.181   0.462   0.970   0.001   0.030
B F  1HE8A                413    0.068  13.708   0.132   0.970   0.001   0.030
E A  1HE8A                414    0.299  32.950   0.774   0.970   0.001   0.030
E V  1HE8A                415    0.472  72.485   1.543   0.970   0.001   0.030
B I  1HE8A                416    0.055  10.249   0.778   0.970   0.001   0.030
B L  1HE8A                417    0.060  11.078   0.535   0.970   0.001   0.030
E E  1HE8A                418    0.399  69.635   1.095   0.970   0.001   0.030
E A  1HE8A                419    0.375  41.292   1.426   0.970   0.001   0.030
B Y  1HE8A                420    0.048  10.300   0.420   0.970   0.001   0.030
B L  1HE8A                421    0.209  38.195   0.302   0.923   0.002   0.076
E R  1HE8A                422    0.692 158.468   0.960   0.782   0.003   0.216
E G  1HE8A                423    0.507  39.869   0.475   0.502   0.002   0.495
B C  1HE8A                424    0.180  25.300  -0.157   0.246   0.004   0.750
E G  1HE8A                425    0.506  39.846  -0.327   0.176   0.004   0.820
E T  1HE8A                426    0.691  95.897   0.486   0.694   0.003   0.303
E A  1HE8A                427    0.580  63.883   0.474   0.858   0.002   0.139
B M  1HE8A                428    0.140  28.114  -0.453   0.858   0.002   0.139
E L  1HE8A                429    0.280  51.231   0.998   0.970   0.001   0.030
E H  1HE8A                430    0.628 114.306   1.040   0.970   0.001   0.030
E D  1HE8A                431    0.299  43.115   0.862   0.970   0.001   0.030
B F  1HE8A                432    0.060  12.082   0.660   0.970   0.001   0.030
E T  1HE8A                433    0.441  61.153   0.902   0.970   0.001   0.030
E Q  1HE8A                434    0.433  77.423   1.139   0.970   0.001   0.030
B Q  1HE8A                435    0.114  20.289  -0.808   0.970   0.001   0.030
B V  1HE8A                436    0.239  36.750   0.153   0.970   0.001   0.030
E Q  1HE8A                437    0.494  88.157   1.077   0.970   0.001   0.030
B V  1HE8A                438    0.083  12.834  -0.736   0.970   0.001   0.030
B I  1HE8A                439    0.101  18.685   0.405   0.970   0.001   0.030
E E  1HE8A                440    0.545  95.194   0.588   0.970   0.001   0.030
E M  1HE8A                441    0.403  80.540   1.046   0.970   0.001   0.030
B L  1HE8A                442    0.068  12.396  -0.638   0.970   0.001   0.030
E Q  1HE8A                443    0.314  56.152   0.635   0.970   0.001   0.030
E K  1HE8A                444    0.595 122.392   0.607   0.923   0.002   0.076
B V  1HE8A                445    0.211  32.461  -0.000   0.923   0.002   0.076
B T  1HE8A                446    0.121  16.727  -1.804   0.858   0.002   0.139
E L  1HE8A                447    0.646 118.264  -0.025   0.782   0.003   0.216
E D  1HE8A                448    0.570  82.079   0.850   0.600   0.003   0.397
B I  1HE8A                449    0.083  15.429  -0.665   0.406   0.004   0.590
E K  1HE8A                450    0.533 109.556   0.522   0.246   0.004   0.750
E S  1HE8A                451    0.685  80.282   0.841   0.181   0.016   0.803
B L  1HE8A                452    0.172  31.493  -0.894   0.109   0.005   0.886
E S  1HE8A                453    0.448  52.541   0.062   0.176   0.004   0.820
E S  1HE8A                454    0.620  72.617   0.067   0.694   0.003   0.303
E Q  1HE8A                455    0.603 107.696  -0.262   0.782   0.003   0.216
E V  1HE8A                456    0.387  59.513  -0.115   0.782   0.003   0.216
B I  1HE8A                457    0.220  40.626  -0.115   0.858   0.002   0.139
E S  1HE8A                458    0.642  75.184   0.100   0.923   0.002   0.076
E Q  1HE8A                459    0.522  93.265   0.228   0.858   0.002   0.139
B L  1HE8A                460    0.113  20.672  -0.745   0.858   0.002   0.139
E K  1HE8A                461    0.421  86.517   0.051   0.782   0.003   0.216
E Q  1HE8A                462    0.586 104.695  -0.095   0.782   0.003   0.216
E K  1HE8A                463    0.310  63.808  -0.753   0.782   0.003   0.216
B L  1HE8A                464    0.184  33.617  -1.330   0.694   0.003   0.303
E E  1HE8A                465    0.600 104.785  -0.738   0.694   0.003   0.303
E N  1HE8A                466    0.502  73.507  -0.460   0.600   0.003   0.397
B L  1HE8A                467    0.237  43.340  -1.190   0.502   0.002   0.495
E Q  1HE8A                468    0.618 110.446  -1.157   0.339   0.016   0.645
E N  1HE8A                469    0.577  84.400  -1.109   0.184   0.043   0.773
B S  1HE8A                470    0.263  30.824  -0.925   0.113   0.087   0.800
E Q  1HE8A                471    0.602 107.535  -1.833   0.052   0.084   0.864
E L  1HE8A                472    0.351  64.250  -1.271   0.053   0.043   0.903
E P  1HE8A                473    0.342  48.587  -1.760   0.018   0.019   0.964
E E  1HE8A                474    0.552  96.504  -1.795   0.018   0.019   0.964
E S  1HE8A                475    0.534  62.632  -2.105   0.004   0.085   0.910
B F  1HE8A                476    0.207  41.505  -0.854   0.004   0.420   0.576
E R  1HE8A                477    0.454 103.943  -1.028   0.004   0.514   0.481
B V  1HE8A                478    0.143  21.994  -0.290   0.005   0.262   0.733
E P  1HE8A                479    0.319  45.252  -2.276   0.004   0.085   0.910
E Y  1HE8A                480    0.387  82.745  -2.086   0.005   0.045   0.951
E D  1HE8A                481    0.469  67.641  -0.700   0.005   0.015   0.979
E P  1HE8A                482    0.401  56.888  -1.196   0.018   0.019   0.964
E G  1HE8A                483    0.507  39.917  -1.179   0.020   0.205   0.775
B L  1HE8A                484    0.140  25.561   0.280   0.004   0.616   0.381
E K  1HE8A                485    0.236  48.483   0.437   0.001   0.900   0.099
B A  1HE8A                486    0.082   9.047   0.755   0.001   0.900   0.099
B G  1HE8A                487    0.240  18.872  -0.188   0.001   0.900   0.099
B A  1HE8A                488    0.281  30.988  -0.271   0.001   0.900   0.099
B L  1HE8A                489    0.082  15.051  -0.214   0.001   0.900   0.099
E A  1HE8A                490    0.266  29.280   0.259   0.004   0.514   0.481
E I  1HE8A                491    0.285  52.743   0.264   0.056   0.142   0.802
E E  1HE8A                492    0.738 128.841  -0.101   0.118   0.150   0.732
E K  1HE8A                493    0.421  86.579   0.528   0.135   0.317   0.548
B C  1HE8A                494    0.227  31.871  -0.161   0.079   0.592   0.329
E K  1HE8A                495    0.450  92.627   0.728   0.085   0.775   0.140
E V  1HE8A                496    0.291  44.757  -0.169   0.087   0.683   0.230
B M  1HE8A                497    0.177  35.518   0.980   0.074   0.484   0.442
E A  1HE8A                498    0.577  63.640  -1.107   0.069   0.386   0.545
E S  1HE8A                499    0.488  57.135  -1.017   0.066   0.296   0.638
E K  1HE8A                500    0.444  91.290  -0.942   0.021   0.279   0.699
E K  1HE8A                501    0.402  82.753  -0.094   0.021   0.279   0.699
E K  1HE8A                502    0.359  73.887   0.757   0.021   0.279   0.699
B P  1HE8A                503    0.170  24.123  -0.828   0.021   0.279   0.699
B L  1HE8A                504    0.206  37.719   0.302   0.023   0.655   0.322
B W  1HE8A                505    0.229  55.002   0.706   0.011   0.918   0.071
B L  1HE8A                506    0.039   7.232   0.106   0.006   0.962   0.032
E E  1HE8A                507    0.242  42.225   0.844   0.001   0.959   0.040
B F  1HE8A                508    0.042   8.449   0.012   0.000   0.983   0.017
E K  1HE8A                509    0.277  57.041   0.526   0.000   0.983   0.017
B C  1HE8A                510    0.176  24.654  -0.363   0.001   0.900   0.099
E A  1HE8A                511    0.416  45.887  -0.864   0.003   0.718   0.279
E D  1HE8A                512    0.479  69.024  -0.960   0.005   0.336   0.660
E P  1HE8A                513    0.410  58.136  -1.727   0.019   0.141   0.840
E T  1HE8A                514    0.562  77.894  -1.268   0.020   0.205   0.775
E A  1HE8A                515    0.360  39.639  -0.788   0.020   0.205   0.775
E L  1HE8A                516    0.564 103.213  -0.951   0.019   0.141   0.840
E S  1HE8A                517    0.675  79.133  -0.625   0.018   0.088   0.893
E N  1HE8A                518    0.504  73.786  -1.209   0.018   0.088   0.893
E E  1HE8A                519    0.495  86.476   0.248   0.021   0.279   0.699
E T  1HE8A                520    0.353  48.989   0.611   0.004   0.514   0.481
B I  1HE8A                521    0.108  20.054   1.011   0.018   0.846   0.136
B G  1HE8A                522    0.113   8.917   0.282   0.011   0.918   0.071
B I  1HE8A                523    0.048   8.862   0.536   0.011   0.918   0.071
B I  1HE8A                524    0.045   8.343  -0.822   0.018   0.846   0.136
B F  1HE8A                525    0.091  18.163   0.604   0.023   0.655   0.322
E K  1HE8A                526    0.324  66.688   0.134   0.021   0.279   0.699
E H  1HE8A                527    0.431  78.453  -1.135   0.005   0.045   0.951
E G  1HE8A                528    0.493  38.760  -1.263   0.018   0.019   0.964
E D  1HE8A                529    0.569  82.065  -1.731   0.053   0.005   0.942
E D  1HE8A                530    0.256  36.875  -0.540   0.176   0.004   0.820
B L  1HE8A                531    0.095  17.376   0.547   0.622   0.015   0.363
B R  1HE8A                532    0.271  62.105   0.893   0.622   0.015   0.363
E Q  1HE8A                533    0.276  49.258   0.810   0.858   0.002   0.139
B D  1HE8A                534    0.077  11.110  -1.024   0.923   0.002   0.076
B M  1HE8A                535    0.183  36.718   0.204   0.923   0.002   0.076
B L  1HE8A                536    0.159  29.058   0.888   0.970   0.001   0.030
B I  1HE8A                537    0.028   5.180   1.001   0.970   0.001   0.030
B L  1HE8A                538    0.090  16.406   0.844   0.970   0.001   0.030
B Q  1HE8A                539    0.170  30.451   0.787   0.988   0.000   0.012
B I  1HE8A                540    0.041   7.603   0.153   0.988   0.000   0.012
B L  1HE8A                541    0.053   9.778   0.785   0.988   0.000   0.012
E R  1HE8A                542    0.337  77.081   0.845   0.988   0.000   0.012
B I  1HE8A                543    0.093  17.205   0.166   0.988   0.000   0.012
B M  1HE8A                544    0.029   5.703   0.609   0.988   0.000   0.012
B E  1HE8A                545    0.209  36.530   0.507   0.988   0.000   0.012
E S  1HE8A                546    0.347  40.645   1.505   0.988   0.000   0.012
B I  1HE8A                547    0.042   7.751   0.155   0.970   0.001   0.030
B W  1HE8A                548    0.051  12.265   0.843   0.970   0.001   0.030
E E  1HE8A                549    0.513  89.551   1.395   0.923   0.002   0.076
E T  1HE8A                550    0.629  87.270   0.567   0.694   0.003   0.303
E E  1HE8A                551    0.415  72.501  -0.230   0.176   0.004   0.820
E S  1HE8A                552    0.708  82.931  -0.579   0.003   0.003   0.994
B L  1HE8A                553    0.200  36.693   0.282   0.005   0.015   0.979
E D  1HE8A                554    0.425  61.257   0.180   0.004   0.138   0.858
B L  1HE8A                555    0.034   6.170  -0.506   0.004   0.420   0.576
B C  1HE8A                556    0.172  24.163   0.197   0.002   0.816   0.182
B L  1HE8A                557    0.023   4.230   0.573   0.002   0.816   0.182
B L  1HE8A                558    0.148  27.026   1.272   0.002   0.816   0.182
B P  1HE8A                559    0.171  24.237   0.305   0.004   0.616   0.381
B Y  1HE8A                560    0.058  12.459   0.106   0.004   0.616   0.381
E G  1HE8A                561    0.281  22.123   0.215   0.021   0.756   0.223
B C  1HE8A                562    0.029   4.072   0.139   0.021   0.756   0.223
B I  1HE8A                563    0.069  12.709   0.834   0.023   0.655   0.322
B S  1HE8A                564    0.102  11.990  -0.874   0.023   0.655   0.322
B T  1HE8A                565    0.117  16.256  -0.637   0.021   0.279   0.699
B G  1HE8A                566    0.149  11.766  -0.436   0.018   0.019   0.964
E D  1HE8A                567    0.587  84.515  -0.391   0.018   0.019   0.964
E K  1HE8A                568    0.488 100.299  -0.893   0.018   0.047   0.935
B I  1HE8A                569    0.089  16.539  -0.016   0.019   0.141   0.840
B G  1HE8A                570    0.078   6.146  -2.226   0.021   0.279   0.699
B M  1HE8A                571    0.037   7.384   0.033   0.021   0.756   0.223
B I  1HE8A                572    0.064  11.822   0.263   0.018   0.846   0.136
B E  1HE8A                573    0.178  31.132  -0.806   0.018   0.846   0.136
B I  1HE8A                574    0.216  39.923  -0.134   0.023   0.655   0.322
B V  1HE8A                575    0.109  16.753   0.237   0.019   0.141   0.840
E K  1HE8A                576    0.592 121.795  -1.264   0.018   0.019   0.964
E D  1HE8A                577    0.603  86.820  -1.339   0.016   0.005   0.979
B A  1HE8A                578    0.107  11.769  -1.790   0.257   0.016   0.727
E T  1HE8A                579    0.486  67.380   0.176   0.717   0.014   0.269
B T  1HE8A                580    0.164  22.733   0.007   0.717   0.014   0.269
B I  1HE8A                581    0.070  12.913   0.098   0.970   0.001   0.030
E A  1HE8A                582    0.320  35.231   0.007   0.970   0.001   0.030
E K  1HE8A                583    0.451  92.709   0.790   0.970   0.001   0.030
B I  1HE8A                584    0.051   9.453  -0.062   0.975   0.003   0.022
B Q  1HE8A                585    0.148  26.469   0.213   0.938   0.007   0.055
E Q  1HE8A                586    0.459  81.995   0.209   0.879   0.010   0.111
E S  1HE8A                587    0.456  53.420   0.144   0.782   0.003   0.216
B T  1HE8A                588    0.271  37.588  -0.757   0.694   0.003   0.303
E F  1HE8A                589    0.420  84.374  -1.236   0.622   0.015   0.363
E K  1HE8A                590    0.360  74.073  -1.398   0.406   0.004   0.590
E D  1HE8A                591    0.537  77.382  -1.298   0.406   0.004   0.590
E E  1HE8A                592    0.463  80.834  -1.030   0.694   0.003   0.303
B V  1HE8A                593    0.187  28.711  -0.312   0.802   0.014   0.185
B L  1HE8A                594    0.047   8.551  -0.575   0.879   0.010   0.111
B N  1HE8A                595    0.235  34.477   0.784   0.923   0.002   0.076
E H  1HE8A                596    0.351  63.774   0.551   0.970   0.001   0.030
B W  1HE8A                597    0.073  17.677  -0.055   0.970   0.001   0.030
B L  1HE8A                598    0.065  11.975  -0.620   0.975   0.003   0.022
E K  1HE8A                599    0.397  81.601   0.723   0.938   0.007   0.055
E E  1HE8A                600    0.525  91.717  -0.541   0.694   0.003   0.303
E K  1HE8A                601    0.514 105.709  -0.805   0.246   0.004   0.750
B S  1HE8A                602    0.208  24.331  -2.161   0.016   0.005   0.979
E P  1HE8A                603    0.587  83.338  -1.716   0.016   0.005   0.979
E T  1HE8A                604    0.511  70.834  -1.228   0.003   0.003   0.994
E E  1HE8A                605    0.404  70.544  -1.183   0.321   0.003   0.675
E E  1HE8A                606    0.720 125.714  -1.206   0.782   0.003   0.216
E K  1HE8A                607    0.382  78.619   0.517   0.858   0.002   0.139
B F  1HE8A                608    0.083  16.678  -0.264   0.970   0.001   0.030
E Q  1HE8A                609    0.333  59.527   0.533   0.970   0.001   0.030
E A  1HE8A                610    0.443  48.808   1.124   0.970   0.001   0.030
B A  1HE8A                611    0.074   8.188   0.034   0.970   0.001   0.030
B V  1HE8A                612    0.087  13.418   0.190   0.970   0.001   0.030
E E  1HE8A                613    0.330  57.616   0.178   0.970   0.001   0.030
B R  1HE8A                614    0.134  30.732   0.694   0.970   0.001   0.030
B F  1HE8A                615    0.035   7.065   0.054   0.970   0.001   0.030
B V  1HE8A                616    0.053   8.146  -0.275   0.970   0.001   0.030
B Y  1HE8A                617    0.191  40.881   0.578   0.970   0.001   0.030
B S  1HE8A                618    0.035   4.149  -0.801   0.970   0.001   0.030
B C  1HE8A                619    0.051   7.090   0.688   0.970   0.001   0.030
B A  1HE8A                620    0.049   5.367  -0.227   0.970   0.001   0.030
B G  1HE8A                621    0.048   3.801  -1.490   0.970   0.001   0.030
B Y  1HE8A                622    0.078  16.583   0.265   0.970   0.001   0.030
B C  1HE8A                623    0.037   5.223  -0.683   0.970   0.001   0.030
B V  1HE8A                624    0.047   7.301  -0.341   0.970   0.001   0.030
B A  1HE8A                625    0.054   5.984   0.399   0.970   0.001   0.030
B T  1HE8A                626    0.061   8.530   0.090   0.938   0.007   0.055
B F  1HE8A                627    0.074  14.952   0.436   0.938   0.007   0.055
B V  1HE8A                628    0.045   6.855   0.140   0.879   0.010   0.111
B L  1HE8A                629    0.055  10.052   0.729   0.779   0.100   0.120
B G  1HE8A                630    0.111   8.775  -0.711   0.478   0.309   0.214
B I  1HE8A                631    0.040   7.437   0.144   0.231   0.330   0.439
B G  1HE8A                632    0.147  11.545  -0.095   0.118   0.150   0.732
B D  1HE8A                633    0.170  24.439  -1.456   0.052   0.084   0.864
B R  1HE8A                634    0.203  46.395  -0.164   0.053   0.043   0.903
B H  1HE8A                635    0.217  39.418  -0.362   0.018   0.019   0.964
B N  1HE8A                636    0.286  41.826  -0.813   0.018   0.019   0.964
E D  1HE8A                637    0.376  54.138  -0.823   0.018   0.019   0.964
B N  1HE8A                638    0.118  17.290  -0.892   0.020   0.205   0.775
B I  1HE8A                639    0.047   8.639   0.514   0.021   0.756   0.223
B M  1HE8A                640    0.068  13.687   0.181   0.018   0.846   0.136
B I  1HE8A                641    0.036   6.568   0.119   0.018   0.846   0.136
B T  1HE8A                642    0.145  20.125  -0.071   0.021   0.451   0.528
E E  1HE8A                643    0.550  96.102  -0.497   0.004   0.085   0.910
E T  1HE8A                644    0.470  65.231  -0.724   0.005   0.045   0.951
B G  1HE8A                645    0.161  12.679  -0.615   0.005   0.015   0.979
E N  1HE8A                646    0.320  46.877   0.215   0.004   0.138   0.858
B L  1HE8A                647    0.043   7.782   0.332   0.002   0.816   0.182
B F  1HE8A                648    0.036   7.185   0.546   0.001   0.959   0.040
B H  1HE8A                649    0.092  16.789   0.445   0.001   0.900   0.099
B I  1HE8A                650    0.035   6.549   0.308   0.018   0.846   0.136
B D  1HE8A                651    0.129  18.574  -0.448   0.004   0.616   0.381
B F  1HE8A                652    0.157  31.510  -0.269   0.020   0.205   0.775
E G  1HE8A                653    0.325  25.617  -0.285   0.018   0.047   0.935
E H  1HE8A                654    0.500  91.023  -0.424   0.018   0.047   0.935
E E  1HE8A                655    0.468  81.812  -1.282   0.018   0.088   0.893
E R  1HE8A                656    0.487 111.500   0.114   0.018   0.088   0.893
B V  1HE8A                657    0.283  43.482  -0.138   0.018   0.047   0.935
E P  1HE8A                658    0.354  50.176  -0.538   0.053   0.043   0.903
B F  1HE8A                659    0.150  30.125  -0.118   0.052   0.084   0.864
E V  1HE8A                660    0.368  56.562   1.614   0.113   0.087   0.800
B L  1HE8A                661    0.070  12.872  -0.568   0.354   0.048   0.598
B T  1HE8A                662    0.137  19.057   0.043   0.522   0.016   0.462
E P  1HE8A                663    0.507  71.887   0.731   0.923   0.002   0.076
B D  1HE8A                664    0.255  36.717   0.409   0.923   0.002   0.076
B F  1HE8A                665    0.033   6.643   0.873   0.970   0.001   0.030
B L  1HE8A                666    0.158  28.985   0.353   0.970   0.001   0.030
E F  1HE8A                667    0.417  83.612   0.612   0.975   0.003   0.022
B V  1HE8A                668    0.048   7.439  -0.592   0.923   0.002   0.076
B M  1HE8A                669    0.060  11.966  -0.468   0.879   0.010   0.111
E G  1HE8A                670    0.444  34.974  -0.215   0.622   0.015   0.363
E T  1HE8A                671    0.363  50.293  -1.408   0.339   0.016   0.645
E S  1HE8A                672    0.325  38.125  -0.955   0.115   0.016   0.868
E G  1HE8A                673    0.654  51.454  -2.160   0.016   0.005   0.979
E K  1HE8A                674    0.382  78.577  -1.220   0.018   0.019   0.964
E K  1HE8A                675    0.653 134.343  -0.957   0.016   0.005   0.979
E T  1HE8A                676    0.434  60.265  -1.137   0.053   0.005   0.942
B S  1HE8A                677    0.243  28.515  -1.545   0.257   0.016   0.727
E P  1HE8A                678    0.623  88.375   0.001   0.622   0.015   0.363
B H  1HE8A                679    0.245  44.493   0.205   0.717   0.014   0.269
B F  1HE8A                680    0.083  16.598   0.347   0.879   0.010   0.111
E Q  1HE8A                681    0.515  91.961   1.000   0.970   0.001   0.030
E K  1HE8A                682    0.339  69.691   2.057   0.970   0.001   0.030
B F  1HE8A                683    0.015   3.031   1.320   0.970   0.001   0.030
B Q  1HE8A                684    0.152  27.129   1.373   0.988   0.000   0.012
E D  1HE8A                685    0.521  75.033   1.541   0.988   0.000   0.012
B I  1HE8A                686    0.083  15.373   1.488   0.988   0.000   0.012
B C  1HE8A                687    0.013   1.755   1.680   0.988   0.000   0.012
B V  1HE8A                688    0.148  22.717   0.875   0.988   0.000   0.012
E K  1HE8A                689    0.373  76.829   1.536   0.988   0.000   0.012
B A  1HE8A                690    0.022   2.435   0.850   0.988   0.000   0.012
B Y  1HE8A                691    0.039   8.313   0.195   0.988   0.000   0.012
E L  1HE8A                692    0.291  53.282   1.220   0.988   0.000   0.012
B A  1HE8A                693    0.073   8.034   0.399   0.970   0.001   0.030
B L  1HE8A                694    0.019   3.424   1.181   0.975   0.003   0.022
B R  1HE8A                695    0.193  44.151   1.031   0.923   0.002   0.076
E H  1HE8A                696    0.622 113.178   1.221   0.858   0.002   0.139
E H  1HE8A                697    0.286  52.078   0.224   0.600   0.003   0.397
B T  1HE8A                698    0.074  10.292  -0.049   0.858   0.002   0.139
E N  1HE8A                699    0.548  80.169   0.217   0.923   0.002   0.076
E L  1HE8A                700    0.251  45.995   1.400   0.970   0.001   0.030
B L  1HE8A                701    0.026   4.742   0.656   0.975   0.003   0.022
B I  1HE8A                702    0.077  14.208   0.504   0.970   0.001   0.030
B I  1HE8A                703    0.190  35.150   0.216   0.988   0.000   0.012
B L  1HE8A                704    0.049   8.990   0.710   0.988   0.000   0.012
B F  1HE8A                705    0.045   9.052   0.273   0.970   0.001   0.030
B S  1HE8A                706    0.256  30.062  -0.373   0.970   0.001   0.030
B M  1HE8A                707    0.078  15.668  -0.297   0.923   0.002   0.076
B M  1HE8A                708    0.063  12.646  -0.018   0.782   0.003   0.216
B L  1HE8A                709    0.200  36.602  -0.164   0.600   0.003   0.397
E M  1HE8A                710    0.514 102.831  -0.704   0.321   0.003   0.675
E T  1HE8A                711    0.450  62.360  -0.427   0.053   0.005   0.942
E D  1HE8A                712    0.547  78.808  -1.644   0.003   0.003   0.994
B I  1HE8A                713    0.056  10.397   0.623   0.053   0.005   0.942
E E  1HE8A                714    0.509  88.905   1.165   0.502   0.002   0.495
B Y  1HE8A                715    0.282  60.157   0.558   0.782   0.003   0.216
B I  1HE8A                716    0.077  14.153   0.708   0.923   0.002   0.076
E R  1HE8A                717    0.298  68.288   0.783   0.923   0.002   0.076
E D  1HE8A                718    0.641  92.411   0.528   0.923   0.002   0.076
E A  1HE8A                719    0.357  39.374   0.877   0.782   0.003   0.216
B L  1HE8A                720    0.106  19.354  -0.595   0.502   0.002   0.495
E T  1HE8A                721    0.455  63.095   0.685   0.176   0.004   0.820
B V  1HE8A                722    0.303  46.494  -0.678   0.053   0.005   0.942
E G  1HE8A                723    0.751  59.064  -0.293   0.003   0.003   0.994
B K  1HE8A                724    0.238  48.874   0.275   0.003   0.003   0.994
E N  1HE8A                725    0.540  79.115   0.802   0.003   0.003   0.994
E E  1HE8A                726    0.513  89.674   0.675   0.858   0.002   0.139
E E  1HE8A                727    0.727 127.007   1.453   0.923   0.002   0.076
E D  1HE8A                728    0.484  69.802   1.619   0.970   0.001   0.030
B A  1HE8A                729    0.026   2.832   0.605   0.970   0.001   0.030
E K  1HE8A                730    0.267  55.004   1.732   0.970   0.001   0.030
E K  1HE8A                731    0.566 116.406   1.801   0.970   0.001   0.030
B Y  1HE8A                732    0.262  55.989   2.196   0.970   0.001   0.030
B F  1HE8A                733    0.020   4.114   1.202   0.988   0.000   0.012
E L  1HE8A                734    0.324  59.251   2.056   0.988   0.000   0.012
E D  1HE8A                735    0.547  78.779   1.723   0.988   0.000   0.012
B Q  1HE8A                736    0.172  30.683   1.607   0.988   0.000   0.012
B I  1HE8A                737    0.029   5.439   0.782   0.988   0.000   0.012
E E  1HE8A                738    0.513  89.691   1.984   0.970   0.001   0.030
E V  1HE8A                739    0.527  81.031   1.120   0.975   0.003   0.022
B C  1HE8A                740    0.067   9.365  -0.837   0.923   0.002   0.076
B R  1HE8A                741    0.226  51.869  -0.462   0.782   0.003   0.216
E D  1HE8A                742    0.647  93.247  -0.149   0.600   0.003   0.397
E K  1HE8A                743    0.471  96.946  -0.739   0.406   0.004   0.590
E G  1HE8A                744    0.280  22.012  -0.114   0.455   0.046   0.498
E W  1HE8A                745    0.444 106.830   0.063   0.428   0.171   0.402
E T  1HE8A                746    0.292  40.570  -0.173   0.231   0.330   0.439
E V  1HE8A                747    0.428  65.814   0.281   0.231   0.330   0.439
E Q  1HE8A                748    0.394  70.279   0.417   0.118   0.150   0.732
E F  1HE8A                749    0.483  96.878  -1.155   0.003   0.003   0.994

Explain the output.  Go back.
```

---
